# RETRACTED: Ahmad et al. Mechanisms and Therapeutic Implications of Cell Death Induction by Indole Compounds. *Cancers* 2011, *3*, 2955–2974

**DOI:** 10.3390/cancers18010114

**Published:** 2025-12-30

**Authors:** Aamir Ahmad, Wael A. Sakr, KM Wahidur Rahman

**Affiliations:** Department of Pathology, Karmanos Cancer Institute, Wayne State University School of Medicine, Detroit, MI 48201, USA

The journal retracts the article titled “Mechanisms and therapeutic implications of cell death induction by indole compounds” [1], cited above.

Following publication, concerns were brought to the attention of the Editorial Office regarding the reliability of a number of cited studies contained within this review article [1].

Adhering to our standard procedure, an investigation was conducted that confirmed the presence of a significant number of citations that could not be considered valid in the context of this review. As a result, the Editorial Board have lost confidence in the overall findings and have decided to retract this review article [1] as per MDPI’s retraction policy (https://www.mdpi.com/ethics#_bookmark30).

The authors did not provide a comment on this decision.
